# Biological Stoichiometry Regulates Toxin Production in *Microcystis aeruginosa* (UTEX 2385)

**DOI:** 10.3390/toxins11100601

**Published:** 2019-10-16

**Authors:** Nicole D. Wagner, Felicia S. Osburn, Jingyu Wang, Raegyn B. Taylor, Ashlynn R. Boedecker, C. Kevin Chambliss, Bryan W. Brooks, J. Thad Scott

**Affiliations:** 1 Center for Reservoir and Aquatic Systems Research, Baylor University, Waco, TX 76798, USA; Felicia_Osburn1@baylor.edu (F.S.O.); Jingyu_Wang1@baylor.edu (J.W.); Ashlynn_Boedecker2@baylor.edu (A.R.B.); Kevin_Chambliss@baylor.edu (C.K.C.); Bryan_Brooks@baylor.edu (B.W.B.); Thad_Scott@baylor.edu (J.T.S.); 2 Department of Biology, Baylor University, Waco, TX 76798, USA; 3 The Institute for Ecological, Earth, and Environmental Sciences, Baylor University, Waco, TX 76798, USA; 4 Department of Chemistry and Biochemistry, Baylor University, Waco, TX 76798, USA; Raegyn_Taylor@baylor.edu; 5 Department of Environmental Science, Baylor University, Waco, TX 76798, USA; 6 Institute of Biomedical Studies, Baylor University, Waco, TX 76798, USA

**Keywords:** ecological stoichiometry, nitrogen, phosphorus, HABs, cyanotoxins, microcystin

## Abstract

Harmful algal blooms (HABs) are increasing in magnitude, frequency, and duration globally. Even though a limited number of phytoplankton species can be toxic, they are becoming one of the greatest water quality threats to public health and ecosystems due to their intrinsic toxicity to humans and the numerous interacting factors that undermine HAB forecasting. Here, we show that the carbon:nitrogen:phosphorus (C:N:P) stoichiometry of a common toxic phytoplankton species, *Microcystis*, regulates toxin quotas during blooms through a tradeoff between primary and secondary metabolism. Populations with optimal C:N (< 8) and C:P (< 200) cellular stoichiometry consistently produced more toxins than populations exhibiting stoichiometric plasticity. Phosphorus availability in water exerted a strong control on population biomass and C:P stoichiometry, but N availability exerted a stronger control on toxin quotas by regulating population biomass and C:N:P stoichiometry. Microcystin-LR, like many phytoplankton toxins, is an N-rich secondary metabolite with a C:N stoichiometry that is similar to the optimal growth stoichiometry of *Microcystis*. Thus, N availability relative to P and light provides a dual regulatory mechanism that controls both biomass production and cellular toxin synthesis. Overall, our results provide a quantitative framework for improving forecasting of toxin production during HABs and compelling support for water quality management that limit both N and P inputs from anthropogenic sources.

## 1. Introduction

Humans have altered the environment on a global scale through changes in land use and biogeochemical cycles, causing the numbers and magnitude of harmful algae blooms (HABs) to increase [1]. Harmful algal blooms have been labeled one of the greatest threats to water quality because of their intrinsic ability to produce toxins, and the difficulty in predicting bloom formation and toxicity [2]. A common phytoplankton bloom forming genus, *Microcystis,* has been identified on every continent except Antarctica and is often associated with the production of cyanotoxins such as microcystins [3]. *Microcystis*, like many HABs, are influenced by temperature [4], light [5], and nutrients, particularly nitrogen (N) and phosphorus (P; [6,7]). The relationship among nutrient supply, bloom formation, and microcystin production has been observed in both laboratory and field studies [8,9,10] with some conflicting results, supporting that microcystin production occurs in either low [10], or more optimal N:P availabilities [11]. Furthermore, microcystin synthesis is often correlated with growth rate, with faster growing cells producing more microcystin per cell compared to slower growing cells [12]. With the uncertainty of how nutrient availability affects bloom formation and toxin production, a unifying framework is needed to reveal mechanisms.

Ecological stoichiometry is a framework that could be used to identify mechanisms of bloom formation and cyanotoxin production. This framework explains how elements from the abiotic environment are assimilated into organisms and explores tradeoffs if the supply of elements to the organism is imbalanced from the demand [13]. These tradeoffs could occur through altering primary metabolism causing shifts in the population elemental composition, and/or between primary and secondary metabolism. Toxin production occurs through secondary metabolism with microcystins being synthesized by nonribosmal peptide synthetase [14]. Because microcystins are relatively N-rich compounds with an average C:N of 4.3 by mol (approximately 14% N), low N availability can alter their production [7,15]. Ecological stoichiometry has been applied to examine how microcystins concentration is effected by the carbon (C):N supply of the media in a direct test of the C:nutrient balance hypothesis [7]. When *Microcystis* was grown in N replete conditions, blooms had higher N and microcystin cell quotas compared to N-limited blooms [7]. Additionally, using stoichiometric theory, it is hypothesized that microcystins cell quotas would increase under P-limited conditions [15] as, by definition, N would be in excess. However, a meta-analysis revealed that microcystins cellular quota slightly decreased under P-limited conditions with the excess N possibly being shuttled into N storage molecules [15].

Here, we use experimental bloom populations of *Microcystis aeruginosa* (UTEX 2385) to explore how resource concentrations and ratios of N and P affect its growth, stoichiometry, and the tradeoffs between primary and secondary metabolism affecting growth and microcystin-LR cellular content. Briefly, we performed two different experiments, with one focusing on growth rates and microcystin concentrations using a single P concentration with varied N concentrations, and the second focusing on growth, stoichiometry, and microcystin cell quotas in response to simultaneously variable N and P concentrations. Consistent with stoichiometric theory, we hypothesized that simulated blooms that are nutrient-limited by N or P would alter their cellular physiology to match nutrient supply, causing less biomass to be produced compared to nutrient replete blooms. Additionally, we hypothesized that N requirements for primary metabolism would cause a tradeoff between growth and secondary metabolic toxin production. We therefore predicted *Microcystis* populations supplied with low resource N:P will have lower microcystin cell quotas, less growth, and additively lower total microcystin content compared to high resource N:P populations, irrespective of P concentration.

## 2. Results

### 2.1. Growth and Stoichiometry Experiment

**Figure 1:**
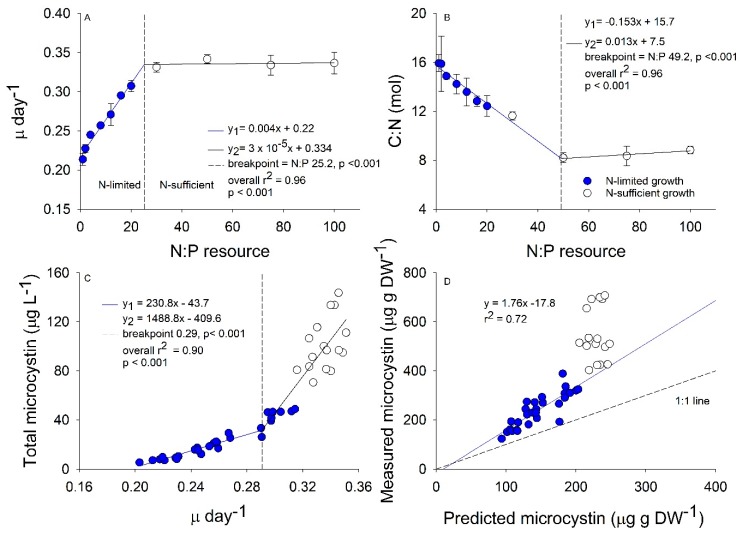
Relationships between experimental resource nitrogen to phosphorus (N:P) by atom with growth rate (**A**) and carbon (C):N stoichiometry (**B**), between measured growth rate (µ day^−1^) with total microcystin-LR concentrations (**C**), and between predicted microcystin concentrations from the growth rate model by Long et al. (2001) against measured microcystin concentration (µg g DW^−1^); (**D**). Colored circles in (**A**–**D**) are classified as N-limited from the relationship in (**A**). Piecewise regressions in (**A**–**C**) indicate where the two linear regressions (y_1_ in blue, y_2_ in black) meet, with breakpoint displayed as vertical dashed line.

Our experimental HAB design revealed a switch between N-limited and N-sufficient conditions at N:P 30 (Figure 1A). The growth rate increased from 0.22 in the N:P 1 to 0.33 at N:P 30 that resulted in a 33% faster growth rate (Figure 1A). The C:N ranged between 16 in the N:P 1 treatment to 8 in all N:P treatments higher than 50 (Figure 1B). Additionally, our temporal analysis revealed that after the first two days of growth after bloom initiation, all of the simulated blooms had similar C:N stoichiometry, and as time progressed, the inorganic N was depleted in culture causing the C:N stoichiometry to increase (Figure S1, Table S1). Total microcystin-LR concentrations best fit a piecewise regression with a break point at a growth rate of 0.29 and a N:P of approximately 16 such that populations with resource N:P < 16 resulted in a shallow slope and populations with resource N:P > 16 had a steeper slope (Figure 1C). The three response variables analyzed resulted in different breakpoints that range between a resource N:P of 16 to 50. We selected the growth rate breakpoint over the C:N or microcystin-LR concentration breakpoints to compare our results with a previously published model for microcystin-LR cellular content by this species [16] that related growth rate directly to microcystin concentration (hereafter referred to as growth rate model). The model was developed based on other previous literature that suggested microcystin content is strongly related to growth/cell division rates [12]. The growth rate model predicts microcystin content increases linearly with growth rate in N-limited *Microcystis* populations. Using this equation, we calculated the predicted concentration in our simulated blooms (Figure 1D). Since our experiment was done as batch populations, the growth rate could be affected by the time the populations remained in stationary phase. This may lead to a decrease in growth rate in the low resource N:P populations; however, blooms with high resource N:P were growing throughout the 12-day experiment. Nitrogen-limited *M. aeruginosa* in our study produced microcystin-LR slightly faster than predicted by the growth rate model with all our data appearing slightly above the 1:1 line and moving farther from the 1:1 with increasing N:P. Nitrogen-sufficient *M. aeruginosa* did not comport with the growth rate model and instead produced microcystin-LR much faster than would be predicted from growth rate alone (Figure 1D).

### 2.2. Interactive Effects of N and P Concentration on Stoichiometry and Toxin Cell Quota

**Figure 2:**
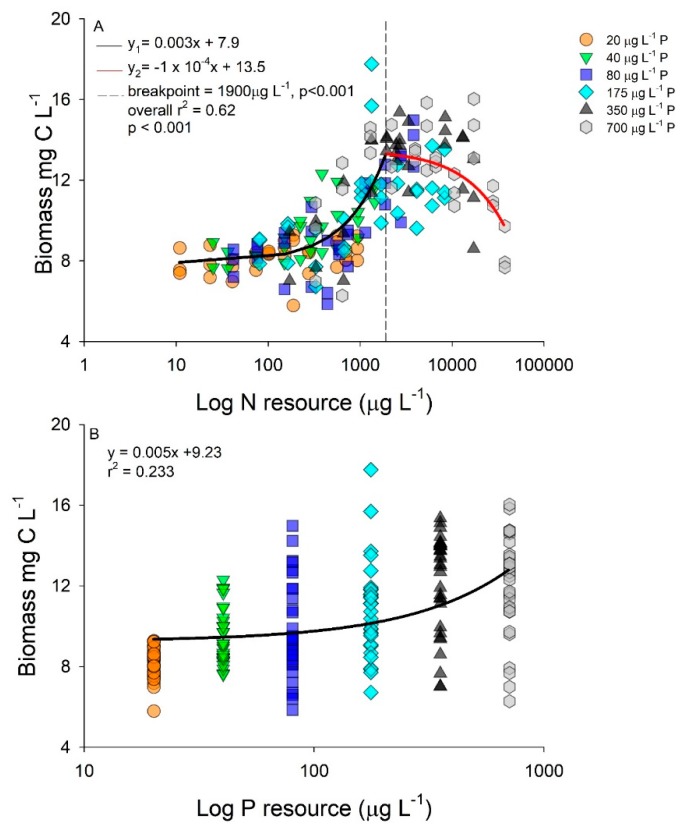
Biomass as carbon (C; mg L^−1^) as a function of log nitrogen (N); (**A**) and phosphorus (P); (**B**) concentrations (µg L^−1^). Regressions performed using all data and color coded by P concentration (20 µg L^−1^ P, orange circle; 40 µg L^−1^ P, green triangle; 80 µg L^−1^ P, blue square; 175 µg L^−1^ P, cyan diamond; 350 µg L^−1^ P, black triangle; and 700 µg L^−1^ P, grey hexagon).

Biomass accumulation was related to both resource N and P concentration, albeit using different models (Figure 2A,B). A piecewise regression was identified with biomass production and log resource N concentrations, with increasing biomass until 1900 µg L^−1^ N-NO_3_ at which point biomass reached a maximum at approximately 13 mg L^−1^ of C (Figure 2A). As resource N concentration increased above 1900 µg L^−1^ N-NO_3,_ a slight decrease in biomass was observed resulting in a negative regression slope after the breakpoint (Figure 2A). Increasing resource P concentrations displayed a positive linear regression slope with the highest biomass accumulation occurring in the highest *p*-level (Figure 2B).

**Figure 3:**
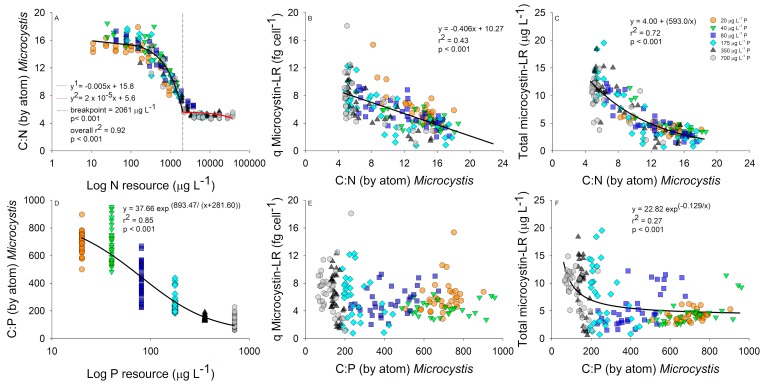
Carbon to nitrogen (C:N) stoichiometry relationships with log resource N concentrations (µg L^−1^); (**A**) and microcystin cell quota (fg cell^−1^); (**B**) and total microcystin-LR (µg L^−1^); (**C**). C:phosphorus (P) stoichiometry relationships with log resource P concentrations (µg L^−1^); (**D**) and microcystin-LR cell quota (fg cell^−1^); (**E**) and total microcystin-LR (µg L^−1^); (**F**). Regressions performed using all data and color coded by P concentration (20 µg L^−1^ P, orange circle; 40 µg L^−1^ P, green triangle; 80 µg L^−1^ P, blue square; 175 µg L^−1^ P, cyan diamond; 350 µg L^−1^ P, black triangle; and 700 µg L^−1^ P, grey hexagon).

Nitrogen resource concentrations were examined against bloom C:N stoichiometry using a piecewise regression. A linear decrease in bloom C:N stoichiometry was observed as the N–NO_3_ concentration increased between 10 and 2061 µg L^−1^, after which the C:N stabilized at an approximate C:N of 5 with higher N concentrations (Figure 3A). Low C:N populations had higher microcystin-LR cell quotas that resulted in a statistically significant negative linear relationship (Figure 3B). However, an exponential decay regression provided a better fit between C:N stoichiometry and total microcystin-LR, which also incorporates the effect of biomass into the relationship, with low C:N blooms producing more total microcystin-LR compared with higher C:N blooms (Figure 3C). The differences between total microcystin-LR and microcystin-LR cell quotas indicate that blooms with optimal C:N stoichiometry overall produced more toxins as the biomass increased. The initial P concentration caused a significant exponential decay relationship with C:P bloom stoichiometry, with the bloom C:P stoichiometry decreasing as the initial P concentration increased (Figure 3D). Comparing bloom C:P stoichiometry to microcystin-LR cell quota resulted in no significant relationships (Figure 3E). However, we did identify an inverse first order polynomial relationship between population C:P stoichiometry and total microcystin-LR further indicating populations with optimal C:P stoichiometry produced more microcystin-LR compared to plastic blooms (Figure 3F).

**Figure 4:**
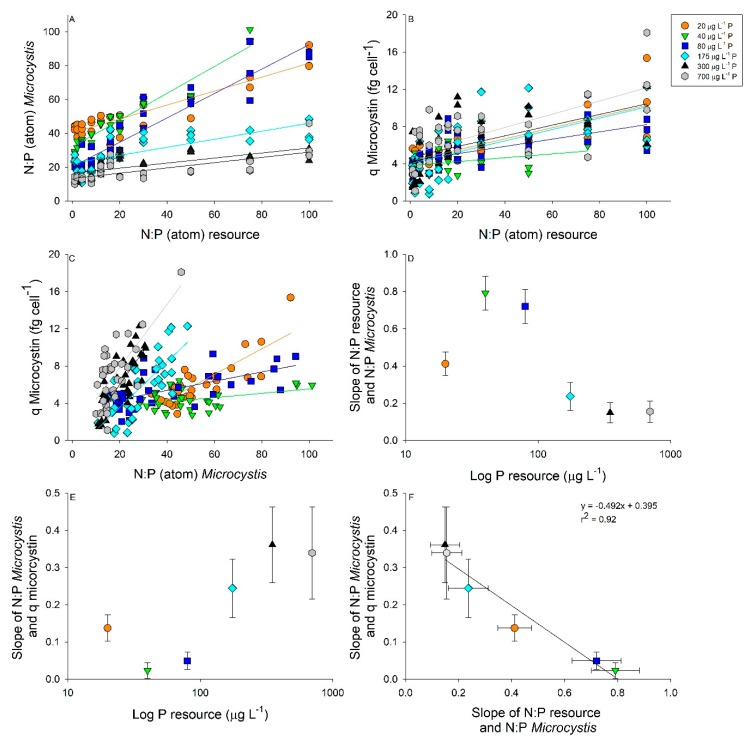
Linear regressions between resource nitrogen to phosphorus (N:P) and *Microcystis* bloom N:P (**A**) and microcystin-LR cell quota (fg cell^−1^); (**B**) separated by P concentration. (20 µg L^−1^ P, orange circle; 40 µg L^−1^ P, green triangle; 80 µg L^−1^ P, blue square; 175 µg L^−1^ P, cyan diamond; 350 µg L^−1^ P, black triangle; and 700 µg L^−1^ P, grey hexagon). Relationships between *Microcystis* N:P and microcystin cell quotas separated by P concentration (**C**). Regression equations and significant differences in slope are found in Table 1. Slopes of resource N:P and *Microcystis* N:P related to resource P concentrations with 95% confidence intervals (**D**). Slopes of *Microcystis* N:P and microcystin cell quota related to resource P concentrations with 95% confidence intervals (**E**). Slopes of resource N:P and *Microcystis* N:P regressed against slopes of *Microcystis* N:P and microcystin cell quota with 95% confidence intervals (**F**).

We then compared how resource N:P was correlated to bloom N:P and microcystin-LR cell quotas separated by P concentrations (Figure 4). Significant linear relationships were found in all *p*-levels between resource N:P and bloom N:P (Figure 4A and Table 1). The lowest 3 *p*-levels (20–80 µg L^−1^ P) had the steepest slopes and displayed the most plasticity within the bloom N:P, whereas higher P concentrations resulted in decreased N:P bloom plasticity (Figure 4A and Table 1). For example, bloom N:P varied between 40 and90 for the 20 µg L^−1^ resource P while the 700 µg L^−1^ resource P varied between 10 and 40 bloom N:P (Figure 4A). Additionally, we found significant linear relationships between the resource N:P and the cell quota of microcystin-LR (Figure 4B). Most regressions had a similar slope, while only 40 µg L^−1^ P had a significantly lower slope compared to the rest of the resource *p*-levels (Figure 4B and Table 1). There were significant linear relationships between *Microcystis* N:P stoichiometry and microcystin-LR cell quotas, with microcystin-LR cell quotas displaying an interaction between resource *p*-level and resource N:P (Figure 4C and Table 1 and Table 2). Irrespective of resource P, blooms with a low resource N:P had lower microcystin-LR cell quotas compared to high resource N:P blooms (Figure 4C). Additionally, microcystin-LR cell quotas were not significantly different in blooms displaying P-limitation (20–80 µg L^−1^ P) from the P-replete blooms (175–700 µg L^−1^ P; Table 2). Examining the slopes from Figure 4A revealed significant differences based on *p*-level with the 20 µg L^−1^ P treatment having a moderate slope that was significantly lower than 40 and 80 µg P L^−1^ and higher than 350 and 700 µg L^−1^ P (Figure 4D and Table 1). The opposite pattern was revealed when comparing the slopes in Figure 4C by *p*-level, with the 40 and 80 µg L^−1^ P having significantly lower slopes compared to the other *p*-levels (Figure 4E and Table 1). Finally, comparing the slopes between resource N:P and the N:P of *Microcystis* with the slopes of *Microcystis* N:P showed that the microcystin-LR cell quota resulted in a strong negative relationship (Figure 4F). This relationship demonstrates that as the plasticity in *Microcystis* N:P stoichiometry increases the microcystin-LR cell quota decreases (Figure 4F).

## 3. Discussion

In accordance with our hypothesis, we identified a tradeoff between primary and secondary metabolism through the biological stoichiometry of *M. aeruginosa* growth and toxin concentration. Blooms that had access to greater N compared to P displayed greater growth and microcystin-LR cell quotas, which in combination resulted in the highest toxin cellular content at the highest N:P ratios. However, blooms that displayed greater elemental plasticity had lower microcystin cell quotas compared to blooms that were more homeostatic. Thus, extremely high resource N:P ratios allowed *M. aeruginosa* to decouple microcystin-LR production from growth and generate more toxin than would have been predicted by growth alone. These results provide a quantitative framework for improving forecasting of microcystin-LR and potentially other toxin production during HABs, and definitively demonstrate that nutrient-rich lakes with high N:P ratios are at the greatest risk for toxic blooms.

Tradeoffs occur in all organisms when experiencing stress and can provide a competitive advantage under certain environmental conditions. For example, it is understood that phytoplankton that can store large amounts of P in highly variable environments will outcompete phytoplankton that cannot when P becomes limiting [17]; however, the energetic cost of this storage could lead to a disadvantage in P-rich conditions. Still, less is known about tradeoffs that occur within a species or organisms. *Microcystis*, like many photoautotrophs, have numerous different secondary metabolic pathways that include microcystin toxin production. A major hypothesis that determines the production of secondary metabolites is the C:nutrient hypothesis that states secondary metabolic products will be synthesized after primary metabolic demands are met [18]. This hypothesis initially seemed promising to explain the production of secondary metabolites; however, it often failed when testing different plant systems under different environmental stressors [18]. Initially, van de Waal et al. [7] found support for this hypothesis for N-rich microcystin production under a range of C:N ratios, with low C:N blooms producing more microcystin. Although, when cyanobacteria are limited by P, the predicted increase in microcystin concentration was not observed [15]. An often overlooked aspect of the C:nutrient balance hypothesis is the energetic and non-C or N elemental demand for synthesizing secondary metabolites.

Currently, it is unknown how much energy is required to synthesize a specific microcystin molecule. Nevertheless, it is a multistep process involving 10 *mcy* genes [19]. Attempts to understand the fitness costs of synthesizing microcystins using both microcystin producing and non-microcystin producing strains [20,21] and microcystin knock-out strains [22] have been performed. Generally, these experiments demonstrate a fitness advantage for the non-toxin producing strains under abiotic stress conditions, including low light and N-limitation. Under optimal conditions, the toxin producing strain displays the fitness advantage by outcompeting the non-toxic strain [21]. While it is unknown how microcystins confers a fitness advantage, it has been hypothesized to be associated with photosynthetic pigments [5] and produced in oxidative stress [23]. Overall, these experiments suggest that a tradeoff exists for microcystins production under suboptimal conditions, even though the mechanism(s) remains elusive.

Our results demonstrate a physiological tradeoff between primary and secondary metabolism by linking elemental stoichiometry to toxin concentration. Specifically, blooms that displayed optimal or low C:P (< 200) and C:N (< 8) had higher microcystin-LR cell quotas than blooms that had a large elemental plasticity. Previous literature has indicated microcystin production is often correlated with N concentrations and is regulated by the global N transcription factor, with greater N concentrations producing more microcystin [15,24]. The global N promoter, NtcA, senses the C and N balance of the cell through the levels of 2-oxoglutarate that couples C and N assimilation, with high concentrations indicating N-stress [25,26,27].

When blooms become N stressed, many of the combined N source transporters are up-regulated and the process of internal N scavenging begins [19,28]. In attempts to rebalance C and N assimilation, photosystem II is down-regulated to decrease C acquisition with excess electrons being transported on to other terminal electron acceptor molecules that can increase oxidative stress [28]. It is possible the oxidative stress and low internal N concentrations causes microcystin production to decrease, favoring primary metabolism until N supply is restored.

If N supply is restored in N-limited blooms, light harvesting pigments are quickly upregulated, or at least no longer down-regulated [19,28]. As the pigment concentration as well as C acquisition is restored, it is possible that other secondary metabolic N storage molecules, like cyanophycin, will be replenished before microcystin synthesis continues as delays in some microcystin gene expression were observed for 24 h after N was restored [19]. This idea of N storage pools being replenished before microcystin synthesis begins is further supported by the rapid accumulation of cyanophycin within 24 h after N supply is restored to N starved cells [29]. In conditions where N is in excess for primary metabolism, we hypothesize N storage molecule pools will be maximized before microcystin synthesis increases and becomes decoupled from growth, thus allowing for increased microcystin concentrations beyond what is predicted from growth. Furthermore, identifying the mechanism(s) of the fitness advantage of microcystin synthesis will help elucidate why excess N can cause microcystin synthesis to decouple from growth.

Besides nutrients effects on toxin cell quotas, both N and P resource concentrations altered biomass accumulation. Our experiments demonstrated a 33% to 50% difference in growth and biomass depending on the resource N and P. Blooms with lower growth rates and biomass demonstrated elemental plasticity with N:P ranging between 10 and 90. This highly plastic range of N:P is much larger than previously described with N:P varying between 11 and 20 with the same resource supply of 1–100 by mol [30]. Furthermore, our results revealed that *Microcystis* blooms can possibly store P, with our population P concentration approaching the resource P supplied (Figure S2). The P content within the cells could either be stored in polyphosphate storage molecules or adsorbed to the cellular membrane [31], and could be used to fuel growth when external P is depleted. The ability of *Microcystis* to possibly store P resulted in low N:P especially in the high resource P concentrations. The biological stoichiometry of these simulated *M. aeruginosa* blooms indicated that the blooms should be classified as N limited using the Redfield ratio [32] or Guildford and Hecky classification [33]. The appearance of imbalance may indicate that *Microcystis* can store P more efficiently compared to N. Thus, only using N:P bloom stoichiometry could give deceptive indicators of nutrient limitation and overall health of HABs.

Our results emphasize toxin quota is maximized during optimal growth conditions as indicated by the media N:P and bloom C:N and C:P. This seems contrary to some field studies that have indicated optimal cyanobacterial biovolume and microcystin concentration in low total N:total P (TN:TP) waters [10,34]. These discrepancies in our results and these field microcystin comparisons could be from differences in classification low vs. high N:P and due to overall trophic status. Both Orihel et al. [34] and Harris et al. [10] used fairly high values of N:P to indicate their boundaries for “low N:P” (20 by mass or 44 by mol in Orihel et al. [34] and 30 by mass or 66 by mol in Harris et al. [10]). Common ratios such as the Redfield ratio (N:P 16:1 by mol) and the lower bound of strictly N-limited lakes (N:P 9:1 by mol) by Guildford and Hecky [33] would drastically change the classifications by Orihel et al. [34] and Harris et al. [10]. Indeed, Scott et al. [11] expanded the analysis by Orihel et al. [34] and found that the microcystin concentration was greatest in Canadian lakes when N:P was 15–20 by mass (33–44 by mol; [11]) and less so in lakes at extremely low and high N:P ratios. Thus, lakes that would be classified as N and P co-limited or balanced growth [33] were at the greatest risk of microcystin-producing blooms, particularly if the concentrations of N and P were also high.

Interestingly, *Microcystis* blooms and microcystin production often occur in eutrophic waters [1] that have fairly low TN:TP caused by biogeochemical processes that favor the accumulation and recycling of P in/from the sediments but the loss of N to the atmosphere via denitrification [35,36]. Nevertheless, continental scale analyses demonstrate the strong N dependence of microcystin production in blooms. Yuan et al. [9] found that the risk of microcystin production increased in US lakes with increasing TN concentrations. Furthermore, the continental-scale Canadian lake data re-analyzed by Scott et al. [11] demonstrated that microcystin production increased significantly above 2600 µg L^−1^ of N. Interestingly, the 2600 µg L^−1^ of N identified as a strong predictor of microcystin in Canadian Lakes, closely matches our results, with the highest biomass accumulation at 1900 µg L^−1^ and optimal C:N at 2061 µg L^−1^ or higher. Thus, our results generally explain the patterns found within natural ecosystems that high microcystin production occurs in waters that are nutrient-rich with optimal or high N:P ratios.

## 4. Conclusions

Our results support the hypothesis that a tradeoff between primary and secondary toxin metabolism occurs and is caused by an imbalance between C and N metabolism in *M. aeruginosa* blooms. Blooms with optimal C:N (< 8) and C:P (< 200) stoichiometry generally accumulated more biomass and had higher microcystin-LR cell quotas. By decreasing resource N and P concentrations, *Microcystis* populations had greater plasticity in N:P stoichiometry, which resulted in decreased microcystin-LR cell quotas. Overall, these results can provide a framework to mechanistically link stoichiometry to bloom toxicity that can aid in the prediction of bloom formation and microcystin-LR and potentially other toxin production. Though our current observations focused on microcystin-LR, future research is needed to determine whether other microcystins and toxins follow such stoichiometric influences. Finally, our results highlight the need for dual N and P management [6] as both N and P controlled the biomass of the bloom, while N disproportionally influenced the toxicity of HABs.

## 5. Materials and Methods

*Microcystis aeruginosa* was purchased from University of Texas at Austin culture collection (UTEX #2385) and maintained in laboratory conditions for over two years. Maintenance growth conditions consisted of transferring 1% of the cell culture into sterilized 0.5x BG-11 media (Sigma-Aldrich, St. Louis, MO, USA C3061) with 1.35 µg L^−1^ of vitamin B_12_ monthly. Cells were then placed into a 26 °C incubator with a light intensity of 100 µE m^−2^ s^−1^ and a light:dark cycle of 14 h:10 h. *Microcystis* cultures used for experiments were grown under maintenance conditions before being transferred to experimental units.

### 5.1. Growth and Stoichiometry Experiment

To determine if tradeoffs between primary metabolism and secondary toxin production occurred within one *p* level, the amount of N as nitrate (NO_3_^−^) in N-free BG-11 (purchased from UTEX) was manipulated. Quadruplicate experimental units were made by adding 5% of 1x N-free BG-11 with 1.35 µg L^−1^ concentration of vitamin B_12_ added for a total volume of 0.85 L in glass jars. Nitrogen treatments were created by varying the NO_3_–N from 0.16 to 16 mg L^−1^ and keeping the P concentration constant (357 µg L^−1^) to give a range of N:P between 1 and 100 (by mol; Table S2), which is commonly found within lakes [36]. We added 1 mg of *M. aeruginosa* as C to initiate the bloom in all experimental units and kept in two incubators held at 26 °C, again with a light intensity of 120 µE m^−2^ s^−1^ and a light:dark cycle of 14 h:10 h. Each day, jars were removed from the incubators, shaken to prevent settling, and a 2 mL subsample was removed to determine *in vivo* fluorescence of chlorophyll (RFU; Turner Designs Trilogy) as an indicator of growth (Figure S3). This aliquot was preserved in Lugol’s iodine to be used for cell counts. Jars were randomly placed back into the incubators to ensure each jar spent equal amount of time in each incubator. In addition to daily RFU measures, we subsampled jars throughout the experiment for particulate C and N stoichiometry after 2, 4–8, 10, and 12 days of growth on 0.7 µm precombusted glass fiber filters (GF/F). On day 12, samples were also collected for particulate and dissolved toxin analysis.

### 5.2. Interactive Effects of N and P Concentration on Stoichiometry and Toxin Cell Quota

To examine interactions between N and P on tradeoffs between growth and microcystin cell quotas, we grew *M. aeruginosa* under 6 different PO_4_–P concentrations ranging 20–700 µg L^−1^ and 11 different NO_3_–N between 0.01 and 36 mg L^−1^ (Table S2) to keep the overall N:P ratio constant between *p*-levels. All 66 treatment combinations were grown in triplicate (total *n* = 198). These conditions were created by supplementing 5% N- and P-free 1x BG-11 with different amounts of N as NaNO_3,_ and P as K_2_HPO_4_ to generate the nominal resource ratios. Additionally, vitamin B_12_ was added at the concentration of 1.35 µg L^−1^ with a total culture volume of 0.85 L. Blooms were initiated by adding 0.9 mg of *M. aeruginosa* as C and placed into 3 identical incubators. To ensure there was no incubator effect, one replicate from each N and P treatment combination was placed into different incubators set at 26 °C, once again with a light intensity of 120 µE m^−2^ s^−1^–140 µE m^−2^ s^−1^ and a light:dark cycle of 14 h:10 h. Each day, the replicate jars were shaken and randomly placed back into the same incubator. This prevented differences in light intensity due to the location of each jar. Every other day, we measured *in vivo* fluorescence of chlorophyll (RFU) to monitor growth and examine for differences between incubators (Figure S4). The experimental blooms were grown for 21 days before being destructively sampled for particulate C, N, P, and microcystin concentrations on 0.7 µm precombusted GF/F filters and the filtrate saved for dissolved microcystin content. This sampling regimen resulted in some populations being in stationary phase for multiple days caused by limiting P and/or N conditions; however, we were more interested in how microcystin concentration varied between nutrient treatments over the same duration of time.

### 5.3. Elemental Composition and Growth Rate

After filtering, samples were stored at −20 °C until analyzed. For C and N analysis, filters were removed from the freezer and placed in to a drying oven at 60 °C for 24 h before analysis on an elemental analyzer (Thermo-Fisher, Flashsmart NC soil, Lakewood, NJ, USA) with the C and N content determined by comparing the peak area obtained to a known aspartic acid standard. These amounts of C and N were then converted back to a concentration within each simulated bloom by correcting for volume filtered. Particulate P filters were analyzed directly from the freezer, following the persulfate digestion molybdate blue colorimetric method [37] using a spectrophotometer at 885 nm wavelength and 1 cm cuvette. We only analyzed particulate P on samples from the second experiment in which resource P concentrations were also manipulated.

We used the amount of biomass accumulated as represented by C over the experiment to determine growth rates in the first experiment using the following equation:

(1) $$µ=\ln\left(\frac{N_{t}}{N_{o}}\right)/t$$

where *N_t_* is the mg L^−1^ of C at the end of the experiment, *N_o_* is the initial mg L^−1^ of C, and *t* is time in days. Carbon was chosen for growth rate calculations because of the strong relationship between C and cell counts (Figure S5).

### 5.4. Cell Counts and Cyanotoxin Analysis

To calculate cell densities, a flow cytometer (BD Diagnostic Systems, FACSVerse, San Jose, CA, USA) using the forward scatter/side scatter clustering was used. Each day prior to use, we quality controlled (QC) the cytometer using standardized beads to check for laser alignment and fluorescence detection. No cell counts were performed until all QC checks were passed. We changed the voltage from the default lyse/wash settings and used a log scale for both the side and forward scatter to obtain a clustered population of cells near the middle of the graph. As the experimental blooms were monocultures, there was only one distinct cluster of cells. These settings were saved to be used for all cell counts. In addition to the standard daily QC test, we also created a stock of preserved *Microcystis* used to ensure the cell counts were similar between runs. This stock had a coefficient of variation of 17% between all runs. Furthermore, we diluted this sample and compared cell counts obtained from the flow cytometer and RFU, that resulted in a strong linear relationship (*r^2^* = 0.9998). Performing these initial trials provided confidence in the cell counts. Blank media without cells resulted in counts between 250 and300 cells mL^-1^. This is less than 0.1% of our lowest cell count within the experiment. For each replicate (*n* = 193), we counted 50,000 cells and used the statistical analysis feature to obtain cells µl^−1^. Out of the 193 samples analyzed, we randomly selected 3 simulated blooms and ran between 6 and13 analytical replicate cell counts, each which resulted in measured coefficients of variation ranging 5–10%.

Dissolved cyanotoxins were quantified using a previously published isotope dilution liquid chromatography mass spectrometry method (LCMS) [38]. *Microcystis aeruginosa* (UTEX 2385) cultures were extracted for polar (saxitoxin, cylindrospermopsin, anatoxin-a) and non-polar (nodularin and microcystin LA, LR, RR, YR, and LY) cyanotoxins, but only microcystin-LR was detected above the method detection limit (MDL; 0.23 ng mg dry weight^−1^, percent recovery 95% ± 1); thus, subsequent extractions were only performed to isolate non-polar cyanotoxins. Isotopically labeled internal standards were used for the analysis of microcystin-LA, LR, RR, and YR. All samples were spiked with internal standards prior to extraction to correct for matrix effect and extraction bias. Previous work has shown absolute matrix effects in fish tissue and water can exceed 50% for microcystins without an isotope dilution method but can be minimized to <10% when using labeled internal standards. Briefly, dissolved samples were filtered through a 0.7 µm GF/F filter, concentrated on HLB cartridges, eluted with acidic methanol, blown dry under N_2_, and reconstituted in 1 mL 90:10 (*v v*^−1^) H_2_O:acetonitrile buffered with 5 mM NH_4_OOCH_3_ and 3.6 mM HCOOH (pH 3.7).

For the first experiment with a single P concentration, particulate samples were prepared by isolating cells through centrifugation at 3500 RPM for 15 min. The cells were lyophilized, weighed on a microbalance, and extracted using the method described below. For the second experiment investigating N and P interaction, cells were collected on a precombusted 0.7 µm GF/F, instead of centrifugation, and then lyophilized on the filters prior to extraction. This method allows for the quantification of microcystin per cell. Lyophilized biomass was spiked with 10 µL of 1 ppm isotopically labeled standard solution and then extracted by adding 1 mL of 75:25 (*v v*^−1^) MeCN:aqueous 0.1% formic acid to the centrifuge tube. The tube was sonicated for 5 min and centrifuged at 3500 rpm for 5 min. The supernatant was collected, and the process repeated two more times, pooling the supernatant each time. The extract was blown dry under N_2_ and reconstituted in 1 mL 90:10 (*v v*^−1^) H_2_O:acetonitrile buffered with 5 mM NH_4_OOCH_3_ and 3.6 mM HCOOH (pH 3.7). All reconstituted samples were syringe filtered into vials for LCMS analysis. Analysis was performed using an Agilent 1260 LC (Agilent Technologies, Santa Clara, CA, USA) coupled to a Agilent G6420 triple quadrupole (Agilent Technologies, Santa Clara, CA, USA) following a previously published method [38]. For both experiments, total microcystin concentration was calculated by converting µg g ^−1^ microcystin to µg L^−1^, assuming the dry weight had a 40% C content and summing it with the dissolved microcystin concentration (experiment 1), or by summing the particulate and dissolved microcystin concentrations directly (experiment 2). Microcystin-LR cell quotas were calculated for samples in experiment 2 by taking the total concentration of microcystin (µg L^−1^) and dividing it by the cells L^−1^.

### 5.5. Statistical Analysis

To determine how growth and stoichiometry is altered by resource N with only one *p*-level, we used a piecewise regression (SigmaPlot, version 13, SyStat Inc, San Jose, CA, USA, 2018). This analysis identifies the point where the two linear regressions meet to be the breakpoint between N-limitation and N-sufficient conditions. The growth rate breakpoint was used for all analyses to separate the N-limited from the N-sufficient treatment. A piecewise regression to examine how the C:N stoichiometry varied with resource N:P was also performed. In addition to the piecewise regression, we performed a repeated measures ANOVA using the stats package in R to test the effect of resource N concentrations on the C:N stoichiometry through time with the replicate as an error term [39].

Total microcystin-LR concentration was regressed against growth rate with a piecewise regression. This model was chosen over an exponential growth curve using the Akaike information criterion (AIC), that states models with a lower AIC provide a better fit to the data. Additionally, we also compared the microcystin-LR concentration to the model concentration predicted by N-limited growth rates as described by Long et al. [16]. We computed a predicted microcystin-LR concentration based on the measured growth rates using

(2) $$MC=\left[\frac{\left(µ\times Q_{MC\;max}\right)-\left(µ\;\times Q_{MC\;min}\right)}{{µ}_{max}}\right]+µ\;\times Q_{MC\;max}$$

where MC is microcystin (mg g^−1^ dry weight), Q_max_ is maximum microcystin concentration (mg g^−1^ dry weight), Q_min_ is minimum microcystin concentration (mg g^−1^ dry weight), and µ is growth rate per day. We compared these predicted microcystin-LR values with our measured values to test the explicit role of growth rate in predicting microcystin concentration across a range of N availability.

To examine the interaction between N and P on growth, stoichiometry, and microcystin-LR concentrations, we pooled all the data from experiment 2 together for comparisons. All regression models were chosen based on AIC values, with lower AIC being the preferred model choice done in SigmaPlot (version 13). We regressed biomass accumulation against resource N and P concentrations to determine how biomass was altered by resource supply. Then, we examined how resource concentrations of N and P affected bloom C:N and C:P, respectively, to observe how plastic the blooms are with different N and P availabilities. Finally, bloom elemental stoichiometry was regressed against microcystin-LR cell quotas and total microcystin-LR concentrations to examine how the bloom effects total toxin concentration and toxin cell quotas.

The data were then separated by the *p*-level to examine how the resource N:P related to cellular N:P and microcystin-LR cell quotas using linear regressions using SigmaPlot (version 13). Slopes of each regression were compared using the program SMATR [40,41]. Differences in slopes would indicate that the N:P stoichiometry and microcystin-LR cell quota of the *Microcystis* would not only depend on the resource ratio, but also the concentration of these resources. Additionally, we regressed cellular N:P stoichiometry against microcystin-LR cell quota separated by *p*-level and compared the slopes using SMATR, with differences in slopes indicating cellular N:P stoichiometry affects microcystin cell quotas. We then used the slopes obtained from the above regressions (Figure 4A,C) and graphed them against resource P concentration with 95% confidence intervals (CI) as error bars. Lastly, the slopes from Figure 4A,C were regressed to examine how bloom stoichiometry effects microcystin-LR production.

## Figures and Tables

**Table 1:** Linear regression for Figure 4 separated by phosphorus (P) concentration with differences between slopes determined using SMATR *p* < 0.05 level as indicated by different letters.

**Regressions for Resource and *Microcystis* N:P as Shown in Figure 4A**				
**P µg L^−1^**	**Equation**	***r*^2^**	***p*-Value**	**Slope Comparisons**
20	y = 0.412x + 40.27	0.84	<0.0001	a
40	y = 0.792x + 31.99	0.92	<0.0001	b
80	y = 0.721x + 20.29	0.89	<0.0001	b
175	y = 0.237x + 22.26	0.56	<0.0001	c
350	y = 0.149x + 16.44	0.49	<0.0001	c
700	y = 0.156x + 13.31	0.48	<0.0001	c
**Regressions for Resource N:P and Microcystin Cell Quota as Shown in Figure 4B**				
**P µg L^−1^**	**Equation**	***r*^2^**	***p*-Value**	**Slope Comparisons**
20	y = 0.060x + 4.29	0.62	<0.0001	a
40	y = 0.021x + 3.81	0.14	0.0232	b
80	y = 0.039x + 4.30	0.39	<0.0001	ab
175	y = 0.061x + 4.03	0.33	0.0003	a
350	y = 0.057 + 4.70	0.35	0.0002	a
700	y = 0.071 + 5.06	0.44	<0.0001	a
**Regressions for *Microcystis* N:P and Microcystin Cell Quota as Shown in Figure 4C**				
**P µg L^−1^**	**Equation**	***r*^2^**	***p*-Value**	**Slope Comparisons**
20	y = 0.137x − 1.15	0.66	<0.0001	a
40	y = 0.023x + 3.20	0.13	0.0322	b
80	y = 0.049x + 3.40	0.36	<0.0001	b
175	y = 0.244x − 1.33	0.55	<0.0001	c
350	y = 0.361x − 1.20	0.62	<0.0001	c
700	y = 0.339x + 1.07	0.49	<0.0001	c

**Table 2:** Two-way analysis of variance (ANOVA) summary table, describing the main effect and interaction between microcystin-LR cell quota and N:P resource, and P concentration. Tukey Post-hoc comparison between different P resource and within a P resource among N:P treatments.

**Factor**				**Df**		***F*-Value**		***p*-Value**			
N:P resource				10		16.83		<0.0001			
P-level				5		6.44		<0.0001			
N:P × P-level				49		1.51		0.035			
Residuals				128							
**Tukey Post Hoc by P Resource**											
	**1**	**2**	**4**	**8**	**12**	**16**	**20**	**30**	**50**	**75**	**100**
20	a	a	a	ab	a	a	a	ab	ab	a	a
40	a	a	a	ab	a	a	a	a	a	a	
80	a	a	a	ab	a	a	a	ab	ab	a	a
175	a	a	a	a	a	a	ab	b	b	a	a
350	a	a	a	ab	a	a	b	ab	b	a	a
700	a	a	a	b	a	a	ab	b	ab	a	a
**Tukey Post Hoc by N:P Resource within P Resource Level**											
	**20**		**40**		**80**		**175**		**350**		**700**
1	a		a		a		ac		a		a
2	a		a		a		abc		a		a
4	ab		a		ab		abc		a		ab
8	a		a		ab		ab		ab		ab
12	ab		a		ab		abc		ab		ab
16	ab		a		ab		abc		ab		ab
20	ab		a		ab		abc		b		ab
30	ab		a		ab		abc		ab		ab
50	ab		a		ab		bc		ab		ab
75	b		a		b		abc		ab		b
100	b				b		abc		b		b

## Supplementary Materials

The following are available online at https://www.mdpi.com/2072-6651/11/10/601/s1, Figure S1: Temporal dynamics of carbon to nitrogen (C:N) by atom across a gradient of resource N: phosphorus (P) by atom generated by altering the resource N while maintaining constant P concentration, Figure S2: Amount of phosphorus (P) within the *Microcystis* blooms compared to P resource concentration for experiment 2, Figure S3: Temporal in vivo chlorophyll-a fluorescence for the growth and stoichiometry experiment, Figure S4: Temporal in vivo chlorophyll-a fluorescence for the phosphorus (P) and nitrogen (N) interaction experiment separated by P-concentration, Figure S5: Relationship between biomass expressed as carbon (mg L^−1^) and cell counts (cells µL^−1^), Table S1: Repeated measures 2-way ANOVA with Tukey’s posthoc test between N:P treatments within a day and between days within a N:P treatment, Table S2: Different nitrogen as nitrate (N-NO_3_) and phosphorus as phosphate (P-PO_4_) concentration in all simulated blooms.
