# Supplementary material for: Biological Stoichiometry Regulates Toxin Production in Microcystis aeruginosa (UTEX 2385)
Source: Toxins (Basel). 2019 Oct 16;11(10):601. doi: 10.3390/toxins11100601 (PMC6833104; doi:10.3390/toxins11100601)
Supplement: Supplementary file 1 [file toxins-11-00601-s001.zip › toxins-610024-supplementary-conversation/toxins-610024-supplementary-conversation.docx]

Supplementary Materials: Biological stoichiometry regulates toxin production in *Microcystis aeruginosa* (UTEX 2385)

Nicole D. Wagner, Felicia S. Osburn, Jingyu Wang, Raegyn B. Taylor, Ashlynn R. Boedecker, C. Kevin Chambliss, Bryan W. Brooks and J. Thad Scott

**Figure S1.** Temporal dynamics of carbon to nitrogen (C:N) by atom across a gradient of resource N: phosphorus (P) by atom generated by altering the resource N while maintaining constant P concentration.

**Figure S2.** Amount of phosphorus (P) within the *Microcystis* blooms compared to P resource concentration for experiment 2.

**Figure S3.** Temporal in vivo chlorophyll-a fluorescence for the growth and stoichiometry experiment.

**Figure S4.** Temporal in vivo chlorophyll-a fluorescence for the phosphorus (P) and nitrogen (N) interaction experiment separated by P-concentration.

**Figure S5.** Relationship between biomass expressed as carbon (mg L^−1^) and cell counts (cells µL^−1^).

**Table S1.** Repeated measures 2-way ANOVA with Tukey’s posthoc test between N:P treatments within a day and between days within a N:P treatment.

| **Variable** | | | **Df** | | | ***F* value** | | | ***p* value** | | |
| --- | --- | --- | --- | --- | --- | --- | --- | --- | --- | --- | --- |
| N:P | | | 10 | | | 441.2 | | | <0.001 | | |
| Day | | | 7 | | | 794.49 | | | <0.001 | | |
| N.P × Day | | | 70 | | | 23.81 | | | <0.001 | | |
| **Post hoc Comparisons** | | | | | | | | | | | |
| **Day** | | | | | | | | | | | |
| **Treatment (N:P)** | **2** | **4** | **5** | **6** | **7** | | **8** | **10** | | **12** | |
| 1 | A | A | A | A | A | | A | A | | A | |
| 2 | A | B | A | B | AB | | A | A | | A | |
| 4 | A | C | B | B | BC | | A | B | | A | |
| 8 | **A** | C | C | C | C | | A | BC | | AB | |
| 12 | A | C | D | D | D | | B | CD | | BC | |
| 16 | A | C | D | D | E | | C | DE | | BC | |
| 20 | A | C | D | D | E | | C | E | | BC | |
| 30 | A | C | D | D | E | | D | F | | C | |
| 50 | A | C | D | D | E | | D | G | | D | |
| 75 | A | C | D | D | E | | D | G | | D | |
| 100 | A | C | D | D | E | | D | G | | D | |
| **Post hoc Comparisons** | | | | | | | | | | | |
| **N:P treatment** | | | | | | | | | | | |
| **Day** | **1** | **2** | **4** | **8** | **12** | **16** | **20** | **30** | **50** | **75** | **100** |
| 2 | A | A | A | A | A | A | ABCD | A | AC | AB | A |
| 4 | AB | A | A | A | AB | A | BD | A | B | A | A |
| 5 | B | AB | B | A | AB | A | ABC | A | BC | A | A |
| 6 | C | B | C | B | B | A | ABD | A | BC | AB | A |
| 7 | C | B | D | C | C | A | ABCD | A | ABC | AB | A |
| 8 | C | B | D | D | D | B | ABCD | A | AC | AB | A |
| 10 | D | C | E | E | E | C | E | B | D | B | B |
| 12 | D | C | E | E | F | D | F | C | E | C | C |

**Table S2.** Different nitrogen as nitrate (N-NO_3_) and phosphorus as phosphate (P-PO_4_) concentration in all simulated blooms.

| **Tradeoff with 1 P concentration** | | | | |
| --- | --- | --- | --- | --- |
| **N:P (mol)** | **PO_4_-P (mg/L)** | **PO_4_-P (mM)** | **NO_3_-N (mg/L)** | **NO_3_-N (mM)** |
| 1 | 0.357 | 0.012 | 0.16 | 0.012 |
| 2 | 0.357 | 0.012 | 0.32 | 0.023 |
| 4 | 0.357 | 0.012 | 0.64 | 0.046 |
| 8 | 0.357 | 0.012 | 1.29 | 0.092 |
| 12 | 0.357 | 0.012 | 1.93 | 0.138 |
| 16 | 0.357 | 0.012 | 2.58 | 0.184 |
| 20 | 0.357 | 0.012 | 3.22 | 0.230 |
| 30 | 0.357 | 0.012 | 4.84 | 0.345 |
| 50 | 0.357 | 0.012 | 8.06 | 0.576 |
| 75 | 0.357 | 0.012 | 12.09 | 0.864 |
| 100 | 0.357 | 0.012 | 16.12 | 1.152 |
| **Tradeoffs with interacting different N and P concentrations** | | | | |
| **N:P (mol)** | **PO_4_-P (mg/L)** | **PO_4_-P (mM)** | **NO_3_-N (mg/L)** | **NO_3_-N (mM)** |
| 1 | 0.02 | 6.4 × 10^−4^ | 0.009 | 6.4 × 10^−4^ |
| 2 | 0.02 | 6.4 × 10^−4^ | 0.018 | 1.2 × 10^−3^ |
| 4 | 0.02 | 6.4 × 10^−4^ | 0.036 | 2.6 × 10^−3^ |
| 8 | 0.02 | 6.4 × 10^−4^ | 0.072 | 5.1 × 10^−3^ |
| 12 | 0.02 | 6.4 × 10^−4^ | 0.108 | 7.7 × 10^−3^ |
| 16 | 0.02 | 6.4 × 10^−4^ | 0.145 | 1.0 × 10^−2^ |
| 20 | 0.02 | 6.4 × 10^−4^ | 0.181 | 1.3 × 10^−2^ |
| 30 | 0.02 | 6.4 × 10^−4^ | 0.271 | 1.9 × 10^−2^ |
| 50 | 0.02 | 6.4 × 10^−4^ | 0.452 | 3.2 × 10^−2^ |
| 75 | 0.02 | 6.4 × 10^−4^ | 0.677 | 4.8 × 10^−2^ |
| 100 | 0.02 | 6.4 × 10^−4^ | 0.903 | 6.5 × 10^−2^ |
| 1 | 0.04 | 1.3 × 10^−3^ | 0.018 | 1.2 × 10^−3^ |
| 2 | 0.04 | 1.3 × 10^−3^ | 0.036 | 2.6 × 10^−3^ |
| 4 | 0.04 | 1.3 × 10^−3^ | 0.072 | 5.1 × 10^−3^ |
| 8 | 0.04 | 1.3 × 10^−3^ | 0.145 | 1.0 × 10^−2^ |
| 12 | 0.04 | 1.3 × 10^−3^ | 0.217 | 1.5 × 10^−2^ |
| 16 | 0.04 | 1.3 × 10^−3^ | 0.289 | 2.1 × 10^−2^ |
| 20 | 0.04 | 1.3 × 10^−3^ | 0.361 | 2.6 × 10^−2^ |
| 30 | 0.04 | 1.3 × 10^−3^ | 0.542 | 3.9 × 10^−2^ |
| 50 | 0.04 | 1.3 × 10^−3^ | 0.903 | 6.5 × 10^−2^ |
| 75 | 0.04 | 1.3 × 10^−3^ | 1.355 | 9.7 × 10^−2^ |
| 100 | 0.04 | 1.3 × 10^−3^ | 1.806 | 0.129 |
| 1 | 0.08 | 2.6 × 10^−3^ | 0.036 | 2.6 × 10^−3^ |
| 2 | 0.08 | 2.6 × 10^−3^ | 0.072 | 5.1 × 10^−3^ |
| 4 | 0.08 | 2.6 × 10^−3^ | 0.145 | 1.0 × 10^−2^ |
| 8 | 0.08 | 2.6 × 10^−3^ | 0.289 | 2.1 × 10^−2^ |
| 12 | 0.08 | 2.6 × 10^−3^ | 0.433 | 3.1 × 10^−2^ |
| 16 | 0.08 | 2.6 × 10^−3^ | 0.578 | 4.1 × 10^−2^ |
| 20 | 0.08 | 2.6 × 10^−3^ | 0.723 | 5.2 × 10^−2^ |
| 30 | 0.08 | 2.6 × 10^−3^ | 1.083 | 7.7 × 10^−2^ |
| 50 | 0.08 | 2.6 × 10^−3^ | 1.806 | 0.129 |
| 75 | 0.08 | 2.6 × 10^−3^ | 2.109 | 0.151 |
| 100 | 0.08 | 2.6 × 10^−3^ | 3.612 | 0.158 |
| 1 | 0.175 | 5.6 × 10^−3^ | 0.079 | 5.6 × 10^−3^ |
| 2 | 0.175 | 5.6 × 10^−3^ | 0.158 | 1.1 × 10^−2^ |
| 4 | 0.175 | 5.6 × 10^−3^ | 0.316 | 2.3 × 10^−2^ |
| 8 | 0.175 | 5.6 × 10^−3^ | 0.632 | 4.5 × 10^−2^ |
| 12 | 0.175 | 5.6 × 10^−3^ | 0.948 | 6.8 × 10^−2^ |
| 16 | 0.175 | 5.6 × 10^−3^ | 1.264 | 9.0 × 10^−2^ |
| 20 | 0.175 | 5.6 × 10^−3^ | 1.580 | 0.113 |
| 30 | 0.175 | 5.6 × 10^−3^ | 2.370 | 0.169 |
| 50 | 0.175 | 5.6 × 10^−3^ | 3.951 | 0.282 |
| 75 | 0.175 | 5.6 × 10^−3^ | 5.927 | 0.423 |
| 100 | 0.175 | 5.6 × 10^−3^ | 7.903 | 0.565 |
| 1 | 0.350 | 0.011 | 0.158 | 1.1 × 10^−2^ |
| 2 | 0.350 | 0.011 | 0.316 | 2.3 × 10^−2^ |
| 4 | 0.350 | 0.011 | 0.632 | 4.5 × 10^−2^ |
| 8 | 0.350 | 0.011 | 1.264 | 9.0 × 10^−2^ |
| 12 | 0.350 | 0.011 | 1.896 | 0.135 |
| 16 | 0.350 | 0.011 | 2.529 | 0.181 |
| 20 | 0.350 | 0.011 | 3.161 | 0.226 |
| 30 | 0.350 | 0.011 | 4.741 | 0.339 |
| 50 | 0.350 | 0.011 | 7.903 | 0.565 |
| 75 | 0.350 | 0.011 | 11.854 | 0.867 |
| 100 | 0.350 | 0.011 | 15.806 | 1.129 |
| 1 | 0.700 | 0.023 | 0.316 | 2.3 × 10^−2^ |
| 2 | 0.700 | 0.023 | 0.632 | 4.5 × 10^−2^ |
| 4 | 0.700 | 0.023 | 1.264 | 9.0 × 10^−2^ |
| 8 | 0.700 | 0.023 | 2.529 | 0.181 |
| 12 | 0.700 | 0.023 | 3.793 | 0.271 |
| 16 | 0.700 | 0.023 | 5.058 | 0.361 |
| 20 | 0.700 | 0.023 | 6.322 | 0.452 |
| 30 | 0.700 | 0.023 | 9.483 | 0.677 |
| 50 | 0.700 | 0.023 | 15.806 | 1.129 |
| 75 | 0.700 | 0.023 | 23.709 | 1.694 |
| 100 | 0.700 | 0.023 | 31.612 | 2.258 |
