# Supplementary material for: Biological Stoichiometry Regulates Toxin Production in Microcystis aeruginosa (UTEX 2385)
Source: Toxins (Basel). 2019 Oct 16;11(10):601. doi: 10.3390/toxins11100601 (PMC6833104; doi:10.3390/toxins11100601)
Supplement: Supplementary file 1 [file toxins-11-00601-s001.zip › toxins-610024-supplementary-conversation/toxins-610024-supplementary-conversation.pdf]

Supplementary Materials: Biological stoichiometry regulates toxin production in *Microcystis aeruginosa* (UTEX 2385)

Nicole D. Wagner, Felicia S. Osburn, Jingyu Wang, Raegyn B. Taylor, Ashlynn R. Boedecker, C. Kevin Chambliss, Bryan W. Brooks and J. Thad Scott

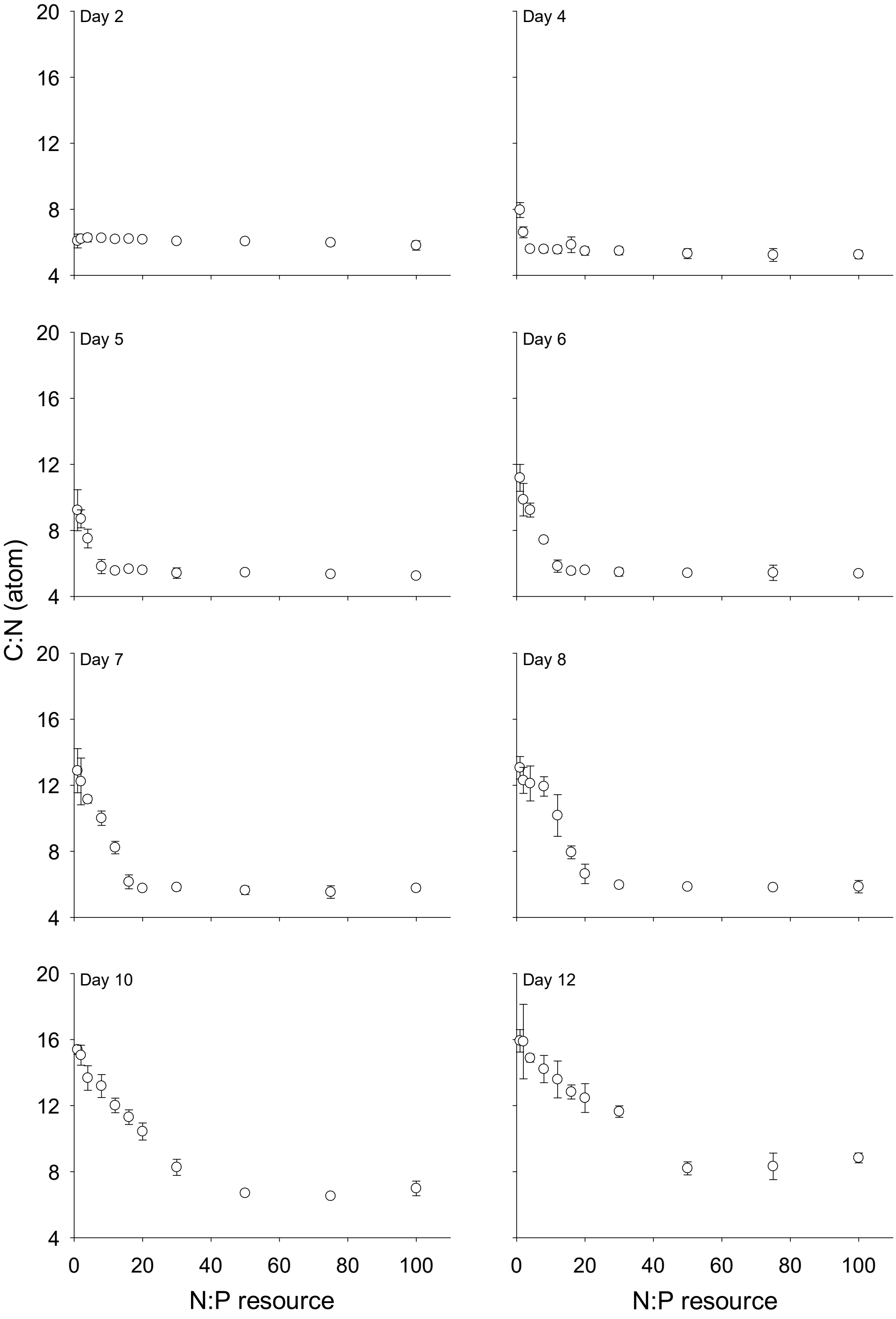

Figure S1. Temporal dynamics of carbon to nitrogen (C:N) by atom across a gradient of resource N: phosphorus (P) by atom generated by altering the resource N while maintaining constant P concentration.

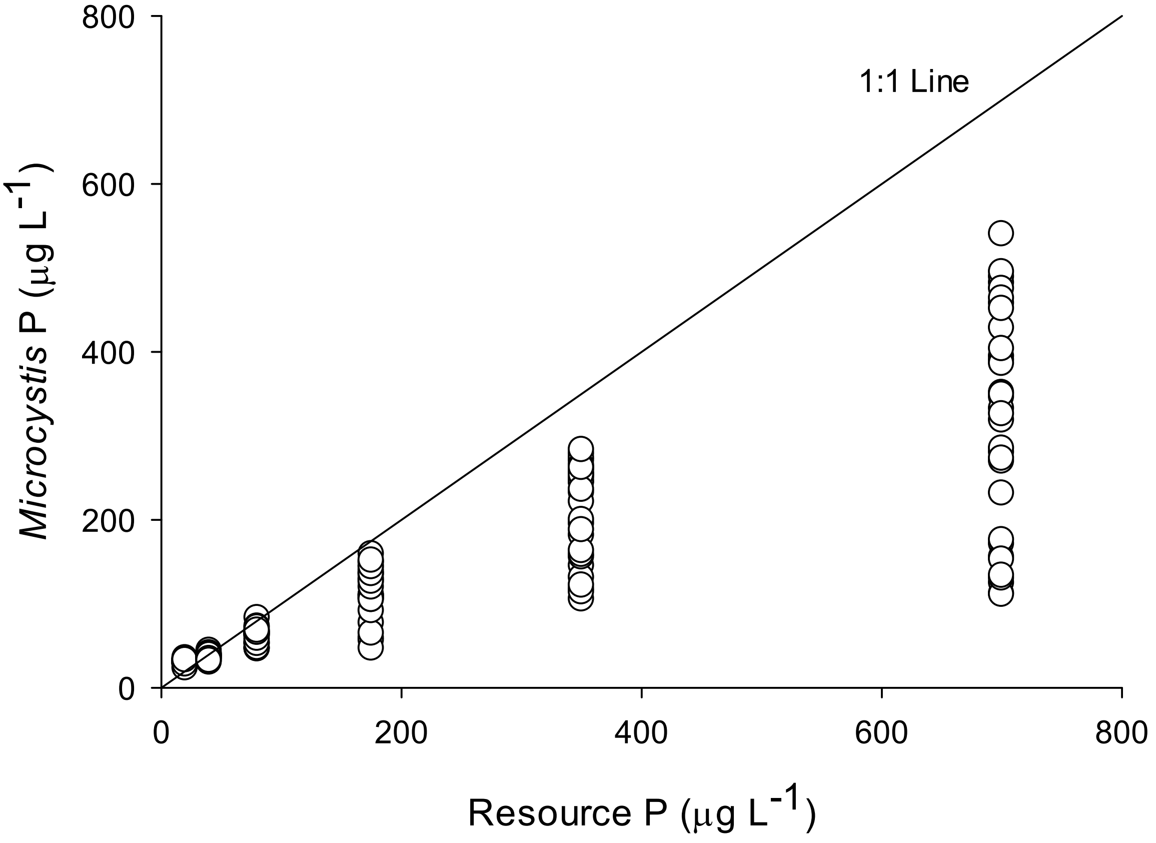

Figure S2. Amount of phosphorus (P) within the *Microcystis* blooms compared to P resource concentration for experiment 2.

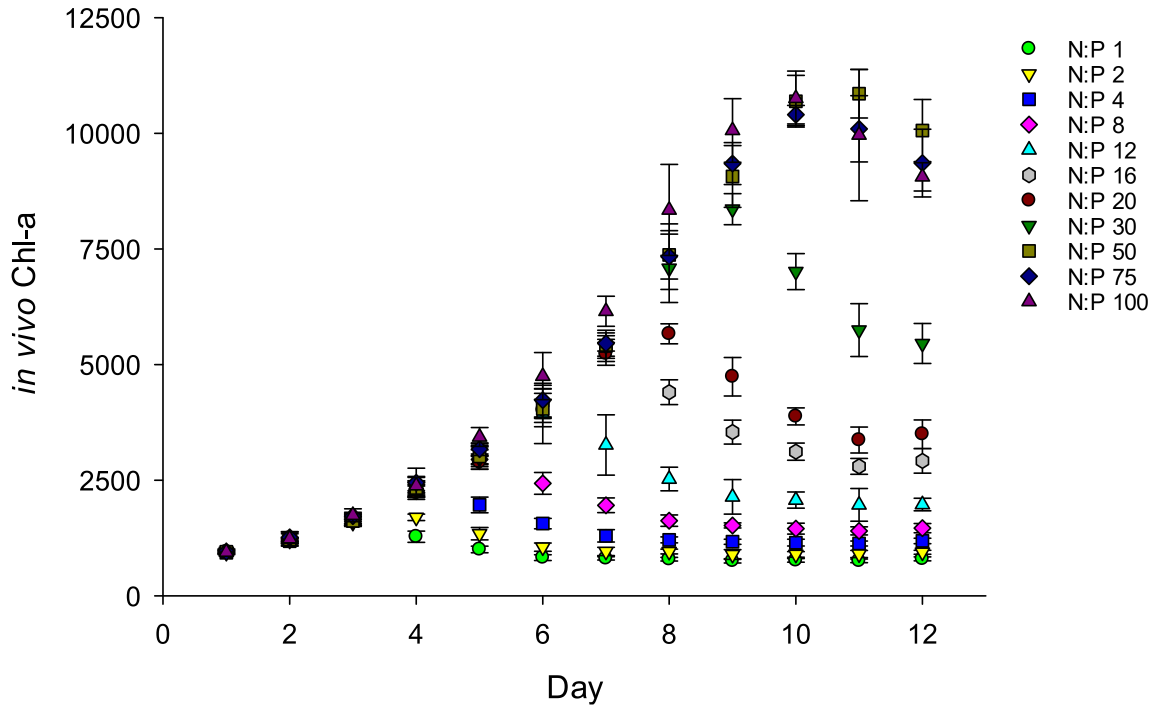

Figure S3. Temporal in vivo chlorophyll-a fluorescence for the growth and stoichiometry experiment.

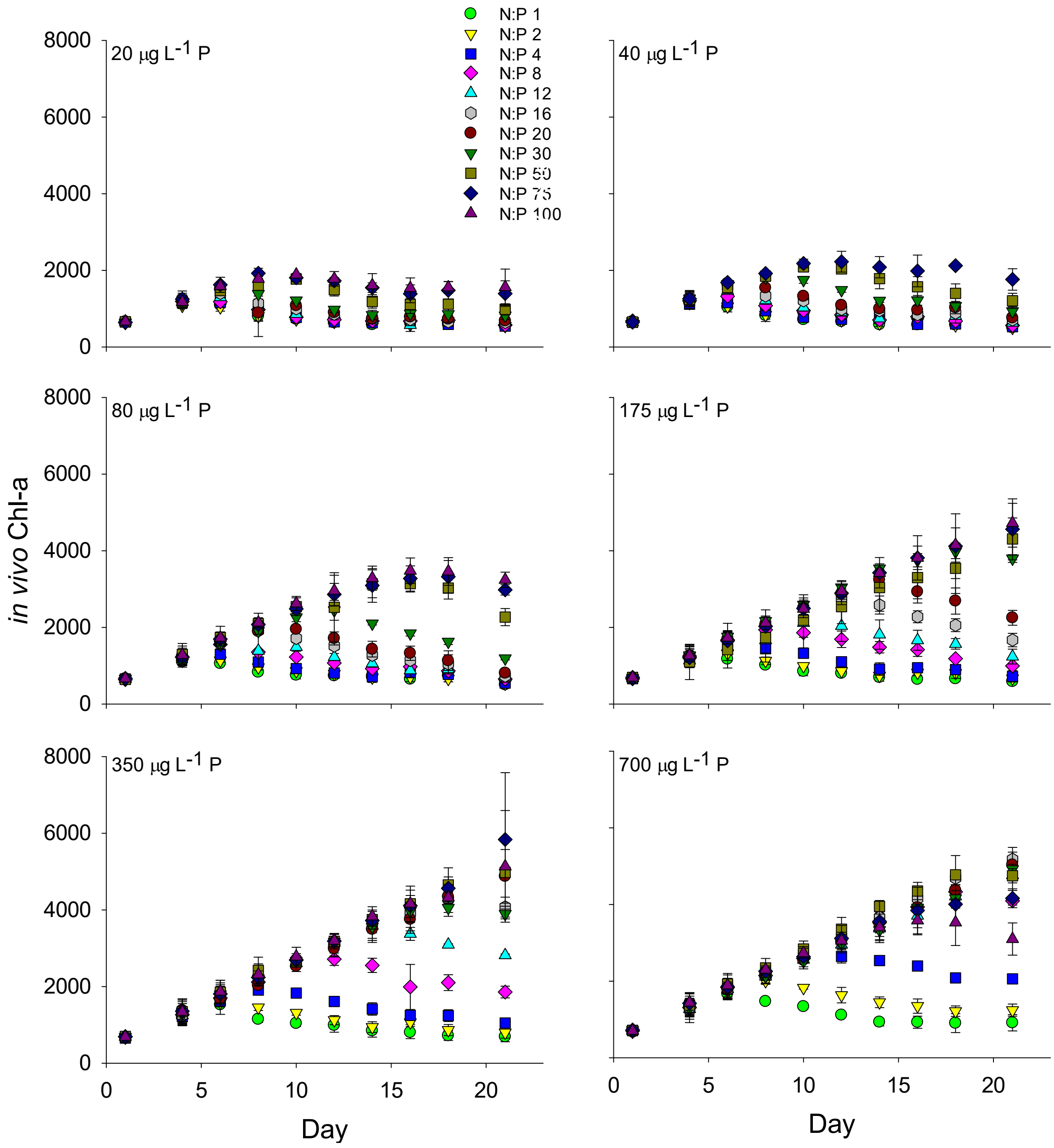

Figure S4. Temporal in vivo chlorophyll-a fluorescence for the phosphorus (P) and nitrogen (N) interaction experiment separated by P-concentration.

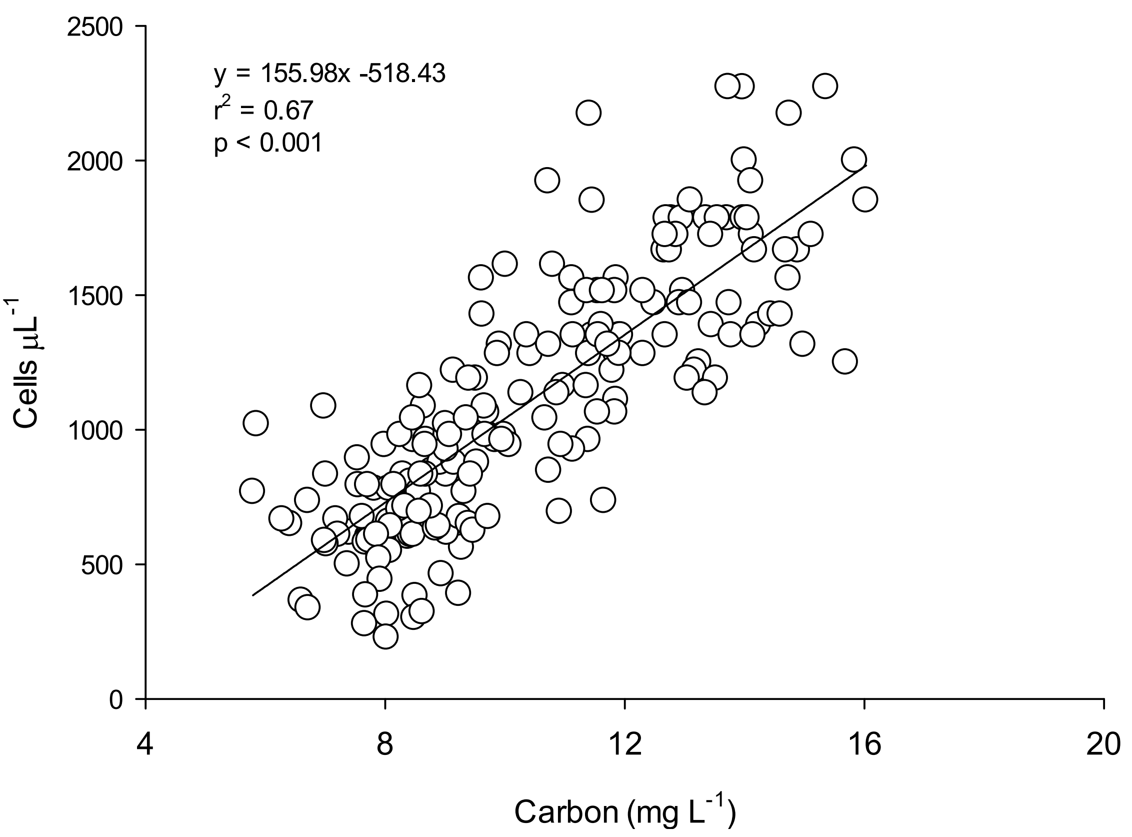

Figure S5. Relationship between biomass expressed as carbon (mg L^{-1}) and cell counts (cells μL^{-1}).

Table S1. Repeated measures 2-way ANOVA with Tukey's posthoc test between N:P treatments within a day and between days within a N:P treatment.

**Variable**	**Df**	***F* value**	***p* value**
N:P	10	441.2	<0.001
Day	7	794.49	<0.001
N.P × Day	70	23.81	<0.001

Post hoc Comparisons

Day

**Treatment (N:P)**	**2**	**4**	**5**	**6**	**7**	**8**	**10**	**12**
1	A	A	A	A	A	A	A	A
2	A	B	A	B	AB	A	A	A
4	A	C	B	B	BC	A	B	A
8	**A**	C	C	C	C	A	BC	AB
12	A	C	D	D	D	B	CD	BC
16	A	C	D	D	E	C	DE	BC
20	A	C	D	D	E	C	E	BC
30	A	C	D	D	E	D	F	C
50	A	C	D	D	E	D	G	D
75	A	C	D	D	E	D	G	D
100	A	C	D	D	E	D	G	D

Post hoc Comparisons

N:P treatment

**Day**	**1**	**2**	**4**	**8**	**12**	**16**	**20**	**30**	**50**	**75**	**100**
2	A	A	A	A	A	A	ABCD	A	AC	AB	A
4	AB	A	A	A	AB	A	BD	A	B	A	A
5	B	AB	B	A	AB	A	ABC	A	BC	A	A
6	C	B	C	B	B	A	ABD	A	BC	AB	A
7	C	B	D	C	C	A	ABCD	A	ABC	AB	A
8	C	B	D	D	D	B	ABCD	A	AC	AB	A
10	D	C	E	E	E	C	E	B	D	B	B
12	D	C	E	E	F	D	F	C	E	C	C

Table S2. Different nitrogen as nitrate (N-NO_3) and phosphorus as phosphate (P-PO_4) concentration in all simulated blooms.

N:P (mol)	PO_4-P (mg/L)	PO_4-P (mM)	NO_3-N (mg/L)	NO_3-N (mM)
**Tradeoff with 1 P concentration**				
1	0.357	0.012	0.16	0.012
2	0.357	0.012	0.32	0.023
4	0.357	0.012	0.64	0.046
8	0.357	0.012	1.29	0.092
12	0.357	0.012	1.93	0.138
16	0.357	0.012	2.58	0.184
20	0.357	0.012	3.22	0.230
30	0.357	0.012	4.84	0.345
50	0.357	0.012	8.06	0.576
75	0.357	0.012	12.09	0.864
100	0.357	0.012	16.12	1.152
**Tradeoffs with interacting different N and P concentrations**				
**N:P (mol)**	**PO_4-P (mg/L)**	**PO_4-P (mM)**	**NO_3-N (mg/L)**	**NO_3-N (mM)**
1	0.02	6.4×10^{-4}	0.009	6.4×10^{-4}
2	0.02	6.4×10^{-4}	0.018	1.2×10^{-3}
4	0.02	6.4×10^{-4}	0.036	2.6×10^{-3}
8	0.02	6.4×10^{-4}	0.072	5.1×10^{-3}
12	0.02	6.4×10^{-4}	0.108	7.7×10^{-3}
16	0.02	6.4×10^{-4}	0.145	1.0×10^{-2}
20	0.02	6.4×10^{-4}	0.181	1.3×10^{-2}
30	0.02	6.4×10^{-4}	0.271	1.9×10^{-2}
50	0.02	6.4×10^{-4}	0.452	3.2×10^{-2}
75	0.02	6.4×10^{-4}	0.677	4.8×10^{-2}
100	0.02	6.4×10^{-4}	0.903	6.5×10^{-2}
1	0.04	1.3×10^{-3}	0.018	1.2×10^{-3}
2	0.04	1.3×10^{-3}	0.036	2.6×10^{-3}
4	0.04	1.3×10^{-3}	0.072	5.1×10^{-3}
8	0.04	1.3×10^{-3}	0.145	1.0×10^{-2}
12	0.04	1.3×10^{-3}	0.217	1.5×10^{-2}
16	0.04	1.3×10^{-3}	0.289	2.1×10^{-2}
20	0.04	1.3×10^{-3}	0.361	2.6×10^{-2}
30	0.04	1.3×10^{-3}	0.542	3.9×10^{-2}
50	0.04	1.3×10^{-3}	0.903	6.5×10^{-2}
75	0.04	1.3×10^{-3}	1.355	9.7×10^{-2}
100	0.04	1.3×10^{-3}	1.806	0.129
1	0.08	2.6×10^{-3}	0.036	2.6×10^{-3}
2	0.08	2.6×10^{-3}	0.072	5.1×10^{-3}
4	0.08	2.6×10^{-3}	0.145	1.0×10^{-2}
8	0.08	2.6×10^{-3}	0.289	2.1×10^{-2}
12	0.08	2.6×10^{-3}	0.433	3.1×10^{-2}
16	0.08	2.6×10^{-3}	0.578	4.1×10^{-2}
20	0.08	2.6×10^{-3}	0.723	5.2×10^{-2}
30	0.08	2.6×10^{-3}	1.083	7.7×10^{-2}
50	0.08	2.6×10^{-3}	1.806	0.129
75	0.08	2.6×10^{-3}	2.109	0.151
100	0.08	2.6×10^{-3}	3.612	0.158
1	0.175	5.6×10^{-3}	0.079	5.6×10^{-3}

2	0.175	5.6×10^{-3}	0.158	1.1×10^{-2}
4	0.175	5.6×10^{-3}	0.316	2.3×10^{-2}
8	0.175	5.6×10^{-3}	0.632	4.5×10^{-2}
12	0.175	5.6×10^{-3}	0.948	6.8×10^{-2}
16	0.175	5.6×10^{-3}	1.264	9.0×10^{-2}
20	0.175	5.6×10^{-3}	1.580	0.113
30	0.175	5.6×10^{-3}	2.370	0.169
50	0.175	5.6×10^{-3}	3.951	0.282
75	0.175	5.6×10^{-3}	5.927	0.423
100	0.175	5.6×10^{-3}	7.903	0.565
1	0.350	0.011	0.158	1.1×10^{-2}
2	0.350	0.011	0.316	2.3×10^{-2}
4	0.350	0.011	0.632	4.5×10^{-2}
8	0.350	0.011	1.264	9.0×10^{-2}
12	0.350	0.011	1.896	0.135
16	0.350	0.011	2.529	0.181
20	0.350	0.011	3.161	0.226
30	0.350	0.011	4.741	0.339
50	0.350	0.011	7.903	0.565
75	0.350	0.011	11.854	0.867
100	0.350	0.011	15.806	1.129
1	0.700	0.023	0.316	2.3×10^{-2}
2	0.700	0.023	0.632	4.5×10^{-2}
4	0.700	0.023	1.264	9.0×10^{-2}
8	0.700	0.023	2.529	0.181
12	0.700	0.023	3.793	0.271
16	0.700	0.023	5.058	0.361
20	0.700	0.023	6.322	0.452
30	0.700	0.023	9.483	0.677
50	0.700	0.023	15.806	1.129
75	0.700	0.023	23.709	1.694
100	0.700	0.023	31.612	2.258
